# Mechanisms of sympathoexcitation *via* P2Y_6_ receptors

**DOI:** 10.3389/fphar.2022.1014284

**Published:** 2022-11-03

**Authors:** Anna Mosshammer, Lifang Zou, Stefan Boehm, Klaus Schicker

**Affiliations:** ^1^ Division of Neurophysiology and Neuropharmacology, Centre of Physiology and Pharmacology, Medical University of Vienna, Vienna, Austria; ^2^ Center of Hematology, The First Affiliated Hospital of Nanchang University, Nanchang, China; ^3^ Clinical Research Center for Hematologic Disease of Jiangxi Province, Nanchang, China

**Keywords:** P2Y_6_ receptor, sympathetic neuron, Ca^2+^-activated Cl-channel, noradrenaline release, excitability

## Abstract

Many drugs used in cardiovascular therapy, such as angiotensin receptor antagonists and beta-blockers, may exert at least some of their actions through effects on the sympathetic nervous system, and this also holds true for e.g., P2Y_12_ antagonists. A new target at the horizon of cardiovascular drugs is the P2Y_6_ receptor which contributes to the development of arteriosclerosis and hypertension. To learn whether P2Y_6_ receptors in the sympathetic nervous system might contribute to actions of respective receptor ligands, responses of sympathetic neurons to P2Y_6_ receptor activation were analyzed in primary cell culture. UDP in a concentration dependent manner caused membrane depolarization and enhanced numbers of action potentials fired in response to current injections. The excitatory action was antagonized by the P2Y_6_ receptor antagonist MRS2578, but not by the P2Y_2_ antagonist AR-C118925XX. UDP raised intracellular Ca^2+^ in the same range of concentrations as it enhanced excitability and elicited inward currents under conditions that favor Cl^−^ conductances, and these were reduced by a blocker of Ca^2+^-activated Cl^−^ channels, CaCCInh-A01. In addition, UDP inhibited currents through K_V_7 channels. The increase in numbers of action potentials caused by UDP was not altered by the K_V_7 channel blocker linopirdine, but was enhanced in low extracellular Cl^−^ and was reduced by CaCCInh-A01 and by an inhibitor of phospholipase C. Moreover, UDP enhanced release of previously incorporated [3H] noradrenaline, and this was augmented in low extracellular Cl^−^ and by linopirdine, but attenuated by CaCCInh-A01. Together, these results reveal sympathoexcitatory actions of P2Y_6_ receptor activation involving Ca^2+^-activated Cl^−^ channels.

## 1 Introduction

Extracellular nucleotides are renowned for being released from all types of cells in a mammalian organism under various physiological and pathophysiological conditions. Accordingly, they are viewed as key signaling molecules in health and disease, and their receptors serve as well-established drug targets in state-of-the art cardiovascular therapy (Vultaggio-Poma et al., 2022). In this respect, a key receptor is the P2Y_12_ subtype which is blocked by several drugs that are routinely employed in the treatment of coronary artery disease and atherothrombotic events. This protein is expressed in the membranes of platelets together with another subtype, P2Y_1_, which may serve as target in anti-platelet therapy as well (Vultaggio-Poma et al., 2022).

Originally, nucleotides were described as signaling elements involved in the control of vascular smooth muscle cells by neurons of the autonomic nervous system (Burnstock, 1972). In fact, the sympathetic nervous system is a major player in the development of cardiovascular diseases (Malpas, 2010). Therefore, it appears prudent to elucidate whether nucleotide receptors that serve as targets for cardiovascular drugs may impact sympathetic neurons. Following this strategy, P2Y_12_ receptors were found to mediate autoinhibition of transmitter release from sympathetic neurons (Lechner et al., 2004), whereas P2Y_1_ receptors mediate an opposing action (Chandaka et al., 2011). In the vasculature, most of the known nucleotide receptors may contribute to the function of smooth muscle, endothelial, and blood cells. In particular, there are P2X1, -2, and -4 as well as P2Y_1_, −2, −6, and −12 receptors on the vascular smooth muscle cells, P2X1, −2, −3, and −4 as well as P2Y_1_, −2, −6, −11, and −12 receptors on endothelial cells, P2X7 and P2Y_13_ receptors on red blood cells, P2X1, and −7 as well as P2Y_1_ and −2 receptors on immune cells, and P2X1 as well as P2Y_1_ and −12 receptors on platelets (Burnstock, 2017). Amongst these, P2Y_6_ receptors have gained increasing attention in the recent past for their role in the development of cardiovascular diseases. This receptor subtype contributes to vascular inflammation (Stachon et al., 2014) which, in turn, induces endothelial P2Y_6_ expression (Riegel et al., 2011). In addition, P2Y_6_ receptors are involved in the signaling of peripheral blood mononuclear cells (Campwala et al., 2014), control arteriolar myogenic tone (Kauffenstein et al., 2016), and heterodimerize with angiotensin AT_1_ receptors to support the development of hypertension (Nishimura et al., 2016). Accordingly, P2Y_6_ receptor antagonists are being developed as novel therapeutic agents to be used in cardiovascular protection (Zhou et al., 2020).

P2Y_6_ receptors belong to the family of P2Y_1_-like receptors which also harbors P2Y_1_, −2, −4, and −11, as opposed to the P2Y_12_-like family with P2Y_12_, −13 and −14, as members. P2Y_6_ receptors are activated by uridine diphosphate (UDP) as natural agonist and most commonly couple to Gq type G proteins (Jacobson et al., 2020; von Kügelgen, 2021). Sympathetic neurons are known to express P2Y_6_ together with P2Y_1_, −2, and −12 receptors (Vartian et al., 2001; Lechner et al., 2004). While P2Y_1_ and -12 receptors mediate facilitation and inhibition of transmitter release from sympathetic neurons, respectively (Lechner et al., 2004; Chandaka et al., 2011), functions of P2Y_2_ and -6 receptors therein have remained comparably unexplored. Yet, uridine nucleotides have been found to promote noradrenaline release from sympathetic neurons through a mechanism involving protein kinase C (PKC), but the receptor involved has remained unidentified (Nörenberg et al., 2000; Vartian et al., 2001).

To elucidate whether P2Y_6_ receptors expressed by sympathetic neurons might contribute to effects of newly developed P2Y_6_ ligands, the present study investigated effects of the P2Y6 agonist UDP and respective antagonists on membrane excitability, ion currents, and noradrenaline release in primary cultures of dissociated rat superior cervical ganglia. This preparation permits an assessment of nucleotide actions arising at sympathetic neurons in isolation and thus independently of target cells such as vascular smooth muscle or endothelial cells as present in, for instance, segments of isolated arteries (Kauffenstein et al., 2016). In such preparations, endogenous nucleotides are released due to neurogenic contractions (Vizi and Sperlágh, 1999) and thereby may contribute to effects observed in response to exogenous drug application.

## 2 Material and methods

### 2.1 Primary cultures of rat superior cervical ganglion neurons

Primary cultures of dissociated rat superior cervical ganglion (SCG) neurons were prepared as described previously (Chandaka et al., 2011). Sprague-Dawley rats (p10-14) were killed by decapitation in full accordance with the rules of the Austrian animal protection and animal experiment law: (https://www.ris.bka.gv.at/Dokumente/BgblAuth/BGBLA_2004_I_118/BGBLA_2004_I_118.html) (https://www.ris.bka.gv.at/Dokumente/BgblAuth/BGBLA_2012_I_114/BGBLA_2012_I_114.html). This procedure does not require an ethical approval according to regulations of the University and the Austrian law. Immediately after decapitation the ganglia were removed, cut into two pieces and incubated in a mixture of collagenase (1.5 mg ml^−1^, Sigma-Aldrich, Vienna, Austria) and dispase (3.0 mg ml^−1^, Roche, Vienna, Austria) for 30 min at 37°C, followed by an incubation in trypsin (0.25%, Worthington, Lakewood, NJ, United States) for 15 min at 37°C. Ganglia were washed twice with calcium free Tyrode solution (in mM: NaCl 150, KCl 4, MgCl2: 2, Glucose 10, Hepes 10, pH 7.4 adjusted with NaOH) and mechanically dissociated *via* trituration in Dulbecco’s modified Eagle’s Medium (DMEM, Sigma-Aldrich, Vienna, Austria) containing 10 mg L^−1^ insulin (Sigma-Aldrich, Vienna, Austria), 50 μg L^−1^ nerve growth factor (R&D Systems Minneapolis MN, United States), 25.000 IU L^−1^ penicillin (Sigma-Aldrich, Vienna, Austria) and 25 mg L^−1^ streptomycin (Sigma-Aldrich, Vienna, Austria). For electrophysiological and calcium imaging experiments, 35,000–50,000 cells were seeded into 0.5 cm diameter glass rings either placed in rat tail collagen coated (Trevigen, Minneapolis MN, United States) 35 mm tissue culture dishes (ThermoFisher scientific, Waltham MA, United States) or collagen coated glass bottom dishes (Mattek, Ashland MA, United States). For release experiments, 45,000 cells were seeded onto 5 mm rat tail collagen coated plastic discs. After 2 h, 5% fetal bovine serum (Biowest, Nuaillé, France) was added and cultures were stored for 4–6 days in a humidified 5% CO_2_ atmosphere at 37°C. Medium was exchanged on the 1st and 4th day after dissociation.

### 2.2 Electrophysiology

All recordings were performed at room temperature (20–24°C) on the somata of single SCG neurons using the perforated patch-clamp technique (Hamill et al., 1981) to avoid dilution of intracellular molecules. Patch pipettes were pulled using a Flaming/Brown Puller (P97, Sutter Instruments, Novato CA, United States) from borosilicate glass capillaries (Science Products, Frankfurt/Main, Germany), front filled with pipette solution and backfilled with the same solution containing 200 μg/ml amphothericin B (Pan Reac AppliChem, Darmstadt, Germany). This routinely resulted in series resistances in the range of 10–20 MΩ 30 min after the establishment of a giga seal, which was compensated for by 60% in voltage clamp experiments. Experiments were performed using an Axopatch 200B patch clamp amplifier (Molecular Devicies, San Jose CA, United States). Current traces were filtered with a 1 kHz 8 pole Bessel filter and digitized at 2 kHz using an Axon Digidata1440 (Molecular Devices, Son Jose CA, United States). Voltage traces were filtered using a 10 kHz Bessel filter and were digitized at 20 kHz. During recordings, cells were continuously superfused using a DAD-12 drug application device (ALA Scientific, NY), which allows a complete exchange of solutions within less than 100 ms (Boehm, 1999).

#### 2.2.1 Measurement of neuronal excitability

Cells were current clamped using a pipette solution consisting of (in mM): K_2_SO_4_ (75); KCl (55); MgCl_2_ (8); HEPES (10); adjusted to pH 7.4 with KOH and a bath solution consisting of (in mM): NaCl (140); glucose (20); HEPES (10); CaCl_2_ (2.5); MgCl_2_ (2); KOH (3); NaOH (2), adjusted to pH 7.4 with NaOH. The calculated liquid junction potential of 8 mV was corrected for at the beginning of the experiment. After measuring the physiological membrane potential (median −73.5 mV; interquartile range (−74.5 to −66.5 mV; see results), it was set to −73 mV by injecting a bias current. For quantification, the traces of membrane potential were averaged during one second periods directly preceding the injection of depolarizing currents (see below).

To asses excitability in SCG neurons, 2 s long current steps (50–300 pA for 2 s in 50 pA increments once every 7 s) were applied and the sum of the elicited action potentials was counted. For experiments with reduced extracellular chloride, 60 mM NaCl was replaced by 60 mM Na-gluconate. To investigate effects of UDP under various conditions (standard conditions, reduced Cl^−^, in the presence of DMSO or channel modulators), current injection was executed first in the absence of the nucleotide and thereafter at the end of a 120 s application of UDP. Whenever the total number of action potentials in the presence of UDP was greater than that determined prior to nucleotide application, this was classified as increase in excitability.

#### 2.2.2 Measurement of chloride currents

Chloride currents were measured by voltage clamping SCG neurons to -70 mV using a bath solution consisting of (in mM): CsCl (130); HEPES (10); glucose (10); MgCl_2_ (2); GdCl_3_ (0.1); tetrodotoxin (0.0005), pH 7.4 adjusted with CsOH and a pipette solution containing (in mM): CsCl (130), TEACl (20), CaCl_2_ (0.24), EGTA (5), glucose (10), HEPES (10), pH 7.2 adjusted with CsOH. UDP was applied again for periods of 120 s. To compensate for current fluctuations occurring independently of UDP, baseline currents were defined as the average of mean current levels during 20 s periods prior to UDP application and mean current levels between seconds 200 and 220 after UDP removal. The UDP induced current was then calculated as difference between this baseline and the mean current level measured between seconds 100 and 120 of UDP application. When such differences in current amplitudes failed to exceed 10 pA, the neurons were classified as non-responders and excluded from further analysis.

#### 2.2.3 Measurement of K_V_7 currents

K_V_7 currents were measured as described before (Ray et al., 2019). Briefly, single SCG neurons were voltage clamped to -30 mV and hyperpolarized to −55 mV for 1 s once every 15 s. K_V_7 currents were defined as the difference between current amplitudes 20 ms after the onset of hyperpolarizations and 20 ms prior to re-depolarization. Solutions were the same as in excitability experiments with the addition of tetrodotoxin (0.5 µM) in the bath solution to block sodium channels. UDP was applied for periods of 120 s, and current amplitudes at the end of such periods were expressed as percentage of current amplitudes determined directly before nucleotide administration (% of control; % inhibition was calculated as 100 - % of control).

### 2.3 Measurement of [^3^H] noradrenaline release

[3H]-noradrenaline uptake and superfusion of SCG neurons was performed as described previously (Chandaka et al., 2011). Culture discs with dissociated SCG neurons were incubated in 0.05 µM [^3^H]noradrenaline (specific activity, 42.6 Ci/mmol; Perkin-Elmer) in culture medium supplemented with 1 mM ascorbic acid at 37°C for 1 h. After labelling, culture discs were transferred to small chambers and superfused with buffer consisting of (in mM): fumaric acid (0.5); Na-pyruvate (5); glucose (20); HEPES (10); ascorbic acid (0.57); NaCl (120); KCl (6); MgCl_2_ (2); CaCl_2_ (2); adjusted to pH 7.4 with NaOH. Superfusion was carried out at 25°C at a rate of 1 ml/min. Modulatory agents, such as linopirdine (30 µM) or CaCCInh-A01 (10 µM), were included in the buffer from minute 50 of superfusion onward. When effects of reductions in extracellular chloride were to be investigated, 60 mM NaCl was replaced by 60 mM Na-gluconate at the very same time point. After a wash out period of 60 min to remove excess radioactivity, 4-min superfusate fractions were collected. UDP (1 µM) was included in the buffer from minute 76 onward.

The rate of spontaneous [^3^H] outflow was obtained by expressing the radioactivity retrieved during a collection period as percentage of the total radioactivity in the cultures at the beginning of this period. The amount of radioactivity in the cultures at the beginning of each collection period is calculated by summing up the radioactivity remaining in the cells at the end of experiments and that retrieved during the respective and all subsequent 4-min collection periods. [^3^H] overflow evoked by UDP was calculated as the difference between the total tritium outflow during the presence of the nucleotide and the estimated basal outflow which was assumed to follow a linear time course throughout experiments. Therefore, basal outflow during the presence of UDP was assumed to equate to the arithmetic mean of the sample preceding UDP inclusion and the last one within each experiment. Differences between total and calculated basal outflow during the first three periods of UDP exposure were expressed as percentages of total radioactivity in the cultures when UDP was added (% of total radioactivity).

### 2.4 AAV production and harvesting

Human Embryonic Kidney cells (HEK-cells 293-LX) were grown in DMEM +10% FBS on 15 cm tissue culture plates to a confluency of 80–90% and transfected with virus plasmids using linear polyethyleneimine (25 KD, PEI; Polysciences Inc., Hirschberg an der Bergstrasse, Germany) as transfection agent. Briefly, 20 µg pHelper, 18 µg pAAV-DJ (Grimm et al., 2008) and 16 µg of pAAV-hSyn-jGCaMP8s (Zhang et al., 2021) plasmids were mixed with DMEM in a final volume of 600 µl. The resulting mixture was combined with a solution consisting of 150 µl PEI (10 µM) and 450 µl DMEM. After an incubation time of 20 min at room temperature, 1.2 ml of DNA/PEI mixture was added to the tissue culture plate. Medium was exchanged after 6 h and virus was harvested after 3 days.

Cells were mechanically detached from the plates and centrifuged for 15 min at 800 g. After centrifugation, the supernatant was discarded and the cell pellet was resuspended in 1 ml of lysis buffer (NaCl, 300 mM; HCl Tris, 50 mM). To ensure virus liberation, the cell suspension was subjected to three freeze thaw cycles, by immersion of the centrifuge tube into a mixture of dry ice and ethanol (96%) for 10 min, followed by a 10 min incubation at 37°C in a water bath and rigorous vortexing. Finally, DNA was removed by incubation with 125 U Benzonase Nuclease (Sigma-Aldrich, Vienna, Austria) for 60 min at 37°C, followed by centrifugation at 5,000 *g* for 15 min. The supernatant was filtered through a 0.45 µM PES membrane filter (Minisart High Flow; Startorius; Goettingen, Germany), and the resulting virus suspension was kept at −80°C for long term storage or at 4°C for up to a month for immediate use. Virus titer was not determined, but transduction efficiency was checked by transducing cells with different amounts of virus and checking visually for jGCaMP8s expression.

### 2.5 Calcium imaging

SCG neurons grown on glass bottom dishes were transduced with hSyn-jGCaMP8s using the AAV described above which ensures GCaMP expression in neurons only. Right after preparation, 4 µl of the virus suspension were added to the dishes and cells were incubated for 24 h. After this, medium was exchanged and calcium imaging experiments were performed 7 days later to ensure proper jGCaMP8s expression. The culture medium was exchanged for imaging buffer (in mM: NaCl, 140; glucose, 20; HEPES, 10; CaCl_2_, 2.5; MgCl_2_, 2; KOH, 3; NaOH, 2, adjusted to pH 7.4) 10–15 min before recording. To inhibit action potential firing during recordings, tretrodotoxin (0.5 µM) was present at all times. Fluorescence imaging was performed on a Nikon Eclipse Ti2 microscope using a 40 × 1.25 NA water immersion objective (Nikon CEE, Vienna, Austria). jGCaMP8s fluorescence was excited using a 470 nm LED (pE4000, Coolled, Andover, United Kingdom) and a GFP Filter (Excitation filter: 470/40 nm, Dichroic: 500 nm, Emission filter 535/50). Images were acquired *via* an EMCCD camera (iXon 888 Ultra; Oxford Instruments, United Kingdom) in 16 bit mode at 10 MHz readout speed with an EM Gain of 40 and an exposure time of 50 ms, at a frequency of 1 frame per second.

To investigate effects of UDP, the nucleotide was applied in increasing concentrations for 60 s each, followed by a wash out of 240 s. The response was measured as the area under the curve (AUC) of the fluorescence signal which was defined as the integral of the baseline corrected calcium reporter signal over the 60 s period of drug application. To compensate baseline fluctuations, baseline fluorescence signals were averaged for 10 s periods prior to UDP application. To correct for differences in reporter expression, the muscarinic receptor agonist oxotremorine-M (OxoM, 10 µM) was applied for 60 s at the end of experiments, and AUC changes caused by UDP were normalized to those of OxoM. Neurons in which the maximum UDP response was found to be less than 20% of that of OxoM were classified as non-responders and excluded from further analysis.

### 2.6 Statistics

In experiments on single cells (electrophysiology and calcium imaging), values are reported as median and interquartile range with *n* values being single cells. In experiments on entire cultures ([^3^H]noradrenaline release) arithmetic means plus standard errors are given. Cultures and cells therein have been obtained from at least three independent preparations. A *p* value of <0.05 was used to determine statistical significance. Statistical significance of differences between two groups was determined by a Mann-Whitney test (for unpaired comparisons of entire cultures) or by a Wilcoxon matched-pairs signed rank test (for paired observations in single cells). For multiple comparisons, a Friedman test followed by Dunn’s multiple comparisons was employed. Figures were prepared using Lualatex and the pgfplots package (https://github.com/pgf-tikz/pgfplots).

### 2.7 Drugs

Uridine diphosphate (UDP), dimethyl sulfoxide (DMSO), linopirdine, (6-(1,1-dimethylethyl)-2-[(2-furanylcarbonyl)amino]-4,5,6,7-tetrahydro-benzo [b]thiophene-3-carboxylic acid (CaCCInh-A01), *N*,*N*″-1,4-Butanediyl*bis* [*N*'-(3-isothiocyanatophenyl)thiourea (MRS2578), 1-[6-[[(17β)-3-Methoxyestra-1,3,5 (10)-trien-17-yl]amino]hexyl]-1*H*-pyrrole-2,5-dione (U73122), 1-[6-[[(17*β*)-3-Methoxyestra-1,3,5 (10)-trien-17-yl]amino]hexyl]-2,5-pyrrolidinedione (U73343), 1,2-bis-(2-aminophenoxy)-ethan-N,N,N′,N′-tetraessigsäure-tetrakis-(acetoxymethyl)-ester (BAPTA-AM), and amphotericin B were obtained from Sigma-Aldrich (Vienna, Austria). Tetrodotoxin (TTX) was purchased from Latoxan (Valence, France). Water-insoluble drugs were first dissolved in DMSO and then diluted in buffer to yield a maximum DMSO concentration of up to 0.3%, which was also included in control solutions.

## 3 Results

### 3.1 Uridine diphosphate depolarizes superior cervical ganglion neurons and increases their firing rate

**FIGURE 1:**
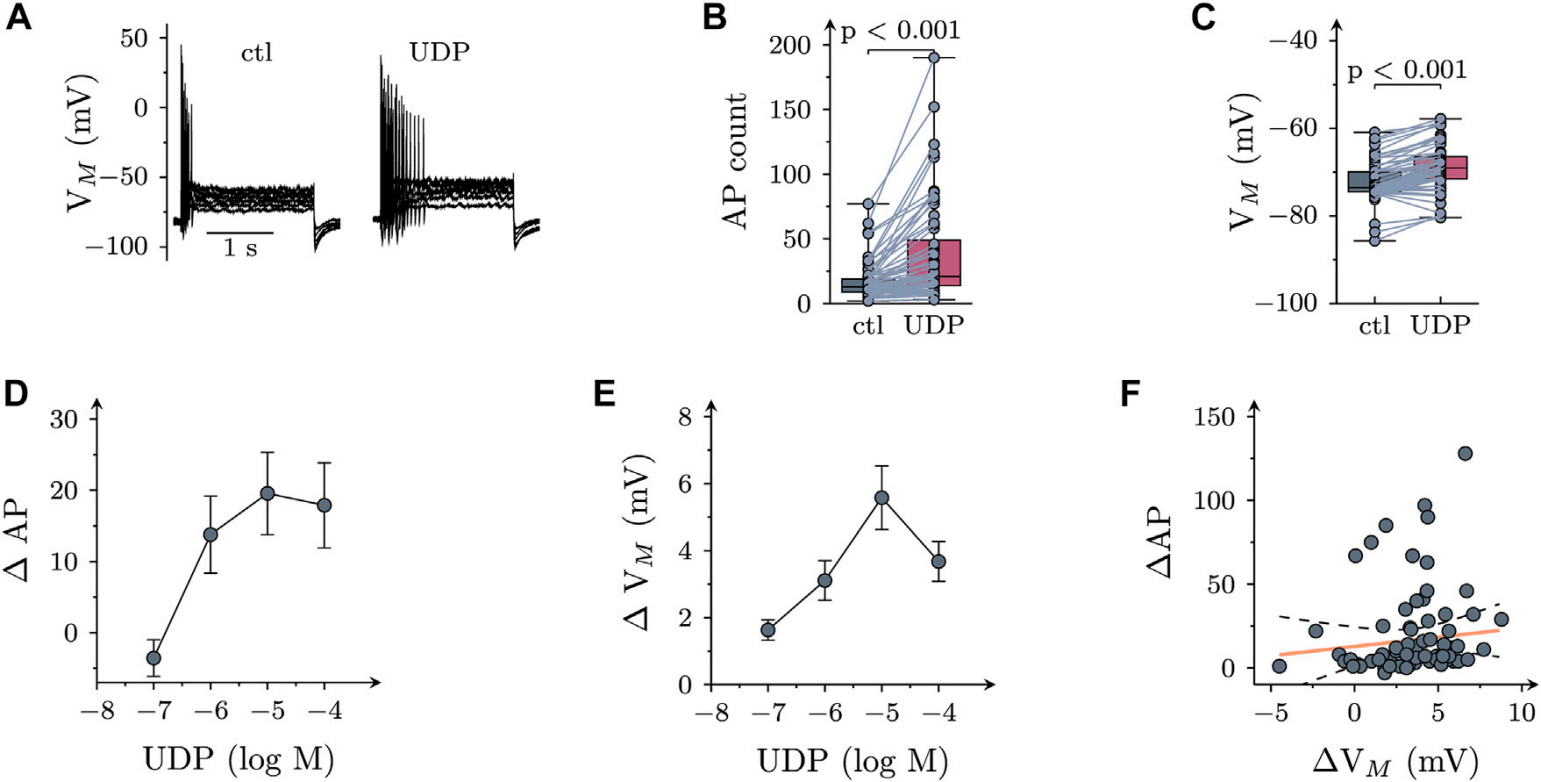
Effects of UDP on sympathetic neuron excitability. Membrane potential was measured in perforated-patch current clamp in single SCG neurons, and 6 depolarizing 2 s currents with amplitudes of 50, 100, 150, 200, 250, and 300 pA were injected to trigger action potentials. **(A)** Original current clamp traces obtained from a SCG neuron before (ctl) and 120 s after application of 10 µM UDP. **(B)** Numbers of action potentials (AP count) fired in response to current injection before (ctl) and during the presence of UDP (10 μM; *n* = 66); *p* values were obtained by a Wilcoxon matched-pairs signed rank test. **(C)** Resting membrane potential (V_m_) determined before (ctl) and during the presence of UDP (10 μM; *n* = 66); *p* values were obtained by a Wilcoxon matched-pairs signed rank test. **(D)** Concentration dependence of changes in action potential firing (Δ AP) in presence of UDP (*n* = 9). **(E)** Concentration dependence of changes in resting membrane potential (Δ V_m_) in presence of UDP (*n* = 9). **(F)** Correlation between changes in resting membrane potential (Δ V_m_) and action potential firing (Δ AP) for the neurons shown in B & C (*n* = 66; *r* ^2^ = 0.022; slope = 1.65 ± 1.38; *p* = 0.24 vs. slope = 0).

The resting membrane potential of SCG neurons amounted to −73.5 (−74.5 to −66.5) mV (*n* = 15). Injection of six depolarizing current pulses (duration 2 s each) with amplitudes increasing from 50 to 300 pA in 50 pA increments resulted in action potential (AP) firing. Under control conditions (Figures 1A,B), a total of 13 (9–19) APs were recorded in response to such current injections. In 66 out of 92 neurons, UDP (10 µM) led to an increase in current-induced APs to 21 (14–49) (Figure 1B) and to a depolarization by 2.2 (1.1–3.3) mV (Figure 1C). In those 26 of 92 neurons that did not shown an increase in action potential firing in the presence of UDP, the nucleotide still led to a depolarization by 1.4 (−0.4–2.6) mV. Both, the increase in AP firing as well as the depolarization caused by UDP were concentration-dependent (Figures 1D,E) with maximal effects achieved at 1–10 µM. Nevertheless, the UDP-dependent increase in AP firing did not appear to be directly related to the extent of membrane depolarization evoked by the nucleotide as the slope of the correlation shown in Figure 1F was not different from zero. Accordingly, all subsequent experiments evaluated changes in excitability by alterations in AP firing only.

### 3.2 Effects of Uridine diphosphate are mediated by P2Y_6_ receptors and involve phospholipase C γ

**FIGURE 2:**
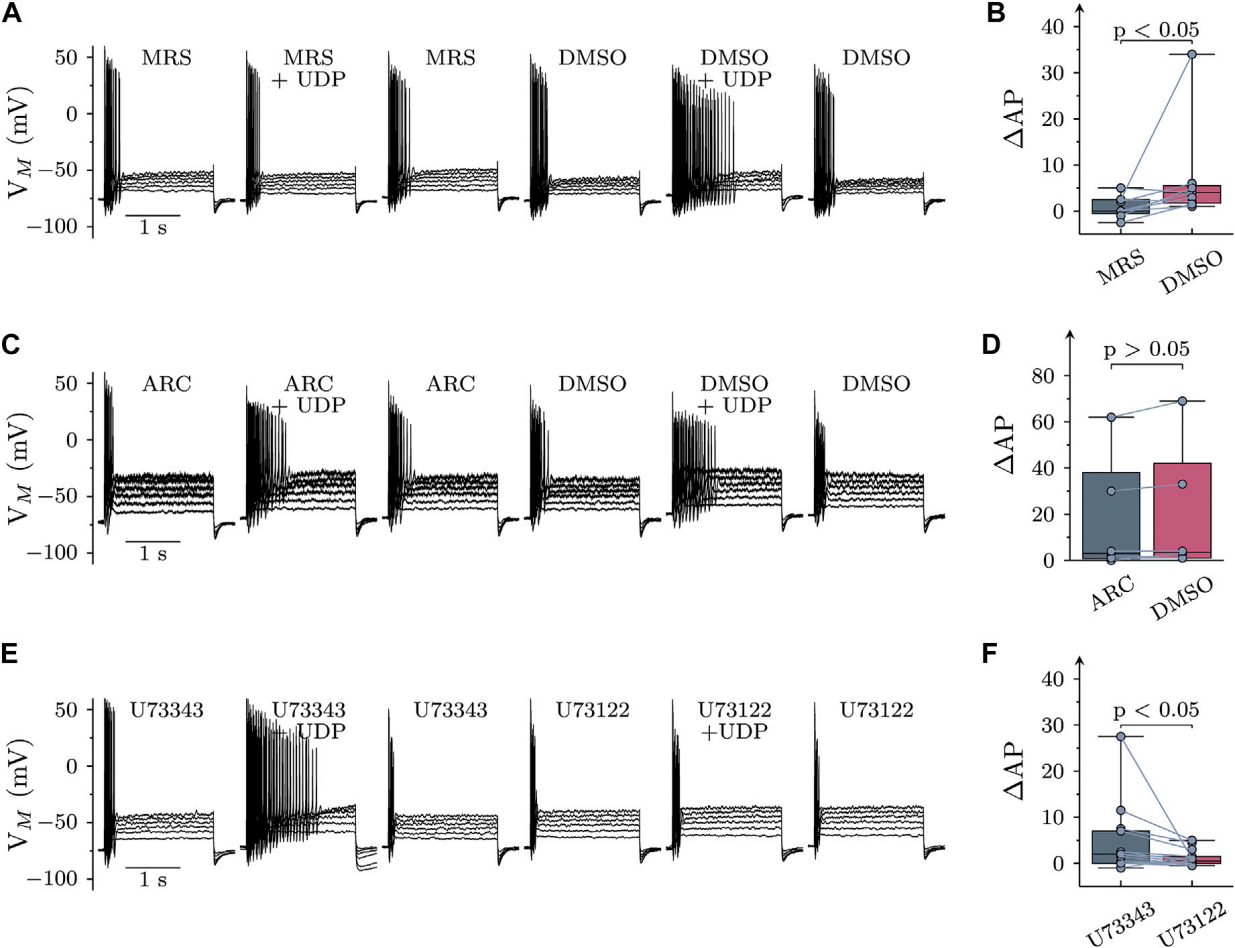
Roles of P2Y receptor subtypes and PLC in the effects of UDP on sympathetic neuron excitability. Membrane potential was measured in perforated-patch current clamp in single SCG neurons, and 6 depolarizing 2 s currents with amplitudes of 50, 100, 150, 200, 250, and 300 pA were injected to trigger action potentials. **(A)** Original current clamp traces were obtained with MRS2578 (MRS; 1 µM in 0.3% DMSO) being present for 60 s before, during, and 300 s after application UDP (1 µM for 120 s); thereafter, MRS2578 was replaced by its solvent (DMSO), and UDP was applied again. **(B)** Changes in AP firing (Δ AP) in response to UDP in either MRS2578 (MRS) or solvent (DMSO; n = 9) as shown in **(A) (C)** Original current clamp traces were obtained with AR-C118925XX (ARC; 1 µM in 0.3% DMSO) being present for 60 s before, during, and 300 s after application UDP (1 µM for 120 s); thereafter, AR-C118925XX was replaced by its solvent (DMSO), and UDP was applied again. **(D)** Changes in AP firing (Δ AP) in response to UDP in either AR-C118925XX (ARC) or solvent (DMSO; *n* = 9) as shown in **(C) (E)** Original current clamp traces were obtained with U73343 (3 µM in 0.1% DMSO) being present for 60 s before, during, and 300 s after application UDP (1 µM for 120 s); thereafter, U73343 was replaced by U73122 (3 µM in 0.1% DMSO), and UDP was applied again. **(F)** Changes in AP firing (Δ AP) in response to UDP in either U73343 or U73122 as shown in E (*n* = 15). Significances of differences were determined by Wilcoxon matched-pair signed rank tests.

SCG neurons express several different P2Y receptors, including P2Y_1_, P2Y_2_, and P2Y_6_ (Vartian et al., 2001). UDP is known as prototypic agonist at P2Y_6_ receptors, and the only other Gq-coupled receptor that might accept UDP as agonist is P2Y_2_ (von Kügelgen, 2006). In order to reveal a role of P2Y_6_ receptors, UDP (1 µM) was applied first in the continuing presence of the specific P2Y_6_ receptor antagonist MRS2578 (1 μM; Mamedova et al., 2004) and thereafter in solvent. In MRS2578, UDP failed to cause major changes in AP firing which became apparent only after removal the antagonist (Figures 2A,B). To exclude a potential contribution of P2Y_2_ receptors, the specific P2Y_2_ antagonist AR-C118925XX (1 μM; Rafehi et al., 2017) was used instead of MRS2578. However, changes in AP firing elicited by UDP (1 µM) were the same whether AR-C118925XX wax present or not (Figures 2C,D). Thus, effects of UDP on sympathetic neuron excitability were mediated by P2Y_6_, but not P2Y_2_ receptors.

P2Y_6_ belongs to the family of P2Y receptors linked to heterotrimeric Gq type G proteins (von Kügelgen, 2006). The prototypic target of G_αq_ subunits is phospholipase C γ (PLC). Therefore, neurons were exposed to the PLC inhibitor U73122 (3 µM), but only after a preceding incubation in its inactive analogue U73343 (3 µM). Increases in AP firing due to the presence of UDP (10 µM) were significantly less in U73122 as compared to U73343 (Figure 2 E & F). This confirms a key role for PLC in raised sympathetic neuron excitability triggered by P2Y_6_ receptor activation.

### 3.3 Uridine diphosphate causes K_V_7 channel inhibition

**FIGURE 3:**
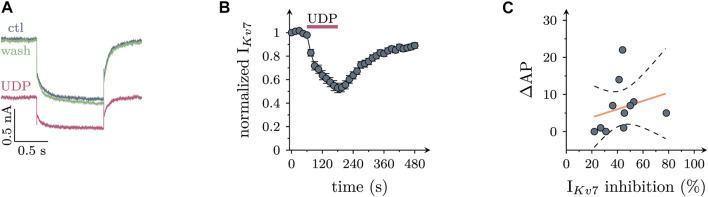
Effects of UDP on currents through K_V_7 channels in sympathetic neurons. Cells were clamped to -30 mV and hyperpolarized to −55 mV for 1 s periods once every 15 s. K_V_7 currents were quantified by the amplitudes of slow current relaxations observed during the 1 s hyperpolarizations. **(A)** Original current traces obtained from a SCG neuron before (ctl), during, and after (wash) the presence of UDP (10 µM). **(B)** Time course of normalized amplitudes obtained before, during, and after the presence of 10 µM UDP as indicated (*n* = 21) **(C)** Correlation between IK_V_7 inhibition and increase in action potential firing (∆AP; *n* = 11; *r* ^2^ = 0.063; slope = 0.11 ± 0.14; *p* = 0.46 vs. slope = 0).

Most, if not all, types of Gq coupled P2Y receptors have been reported to mediate an inhibition of K_V_7 channels in various nerve cells including postganglionic sympathetic neurons (Hussl and Boehm, 2006). To confirm such an action here, neurons were hyperpolarized from −30 mV to −55 mV to deactivate currents through K_V_7 channels. In the presence of 10 µM UDP, resulting current relaxation amplitudes were slowly reduced by up to 50%, and these amplitudes recovered towards baseline even more slowly upon removal of the nucleotide (Figures 3A,B). However, there was no correlation between the extent of UDP-dependent Kv7 current inhibition and the increase in AP firing caused by the nucleotide in the very same neuron (Figure 3C). This hints towards other mechanisms mediating effects of P2Y6 receptor activation on sympathetic neuron excitability.

### 3.4 Uridine diphosphate leads to an increase in intracellular Ca^2+^ and gates calcium activated chloride channels

**FIGURE 4:**
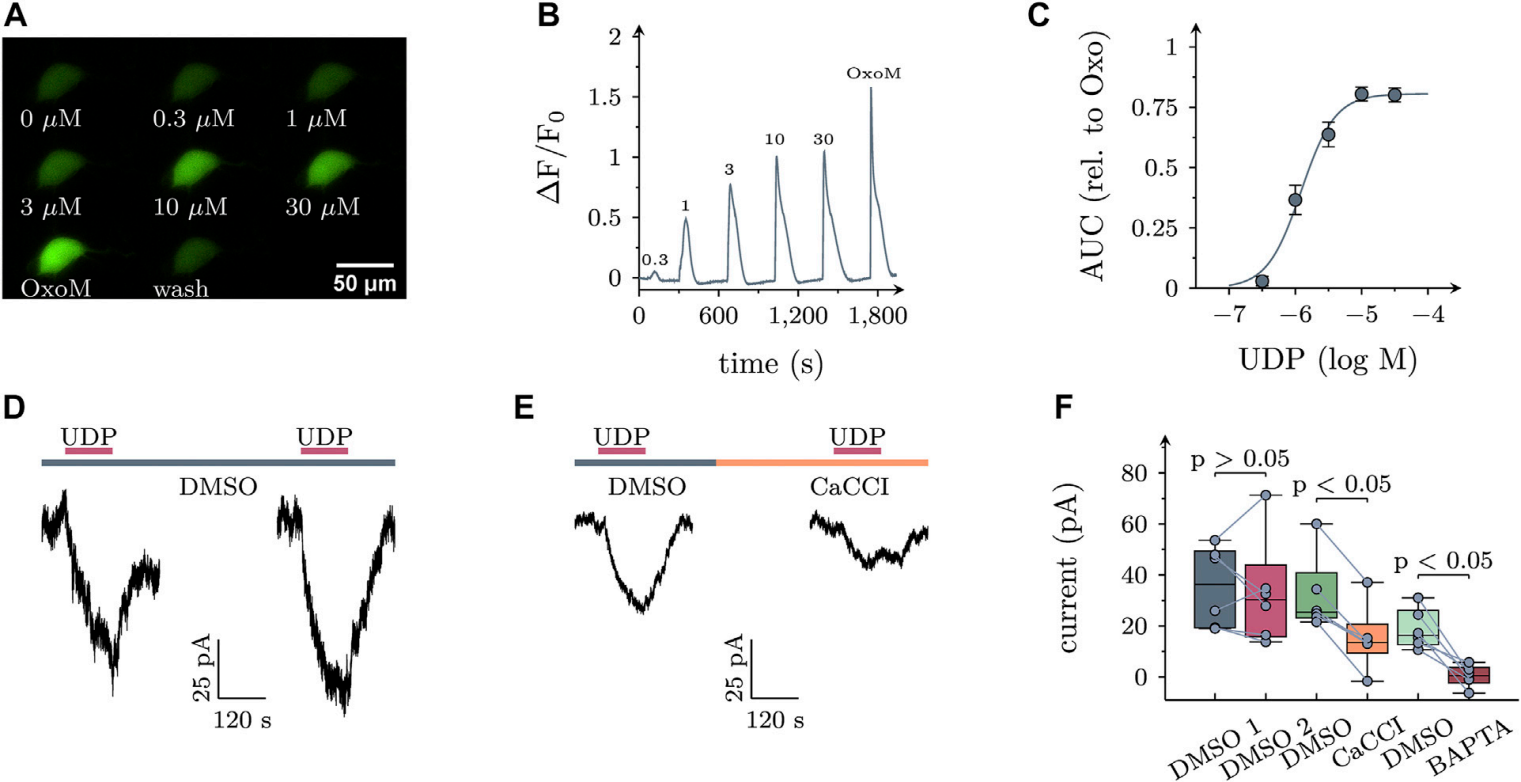
Effects of UDP on intracellular calcium and membrane currents in sympathetic neurons. **(A)** Example images of an SCG neuron expressing jGCaMP8s stimulated with the indicated UDP concentrations or oxotremorine M (OxoM; 10 µM). **(B)** Average time course of jGCaMP8s fluorescence in UDP sensitive cells (*n* = 25). Numbers indicate the applied UDP concentration in µM. **(C)** Concentration dependence of the area under the curve (AUC) of fluorescence traces shown in **(B)** relative to the AUC produced by OxoM (*n* = 25, logEC50 = −5.9+/−0.06, nH = 1.7, *R* ^2^ = 0.67). **(D,E)** Example voltage clamp recordings of UDP induced currents. **(D)** UDP (1 µM) was applied twice, interleaved by a 300 s application of the vehicle control DMSO (0.1%). **(E)** UDP was applied in the presence of the vehicle control DMSO (0.1%) followed by a 300 s application of the specific calcium activated chloride channel inhibitor CaCCInh-A01 (10 µM in 0.1% DMSO) and a second UDP application **(F)** Statistical analysis of experiments depicted in (D & E; DMSO 1: 36.3 (30); DMSO 2: 30.2 (55.5), *n* = 6; DMSO: 25.3 (17.7), CaCCI: 13.5 (11.3), *n* = 6). In addition, after the first UDP application, neurons were exposed to 10 µM BAPTA-AM for 30 min and UDP was applied again in BAPTA-AM [DMSO: 16.3 (13.4), BAPTA-AM 0.5 (6.0)]. *p* values are indicated above the boxes (Wilcoxon matched-pair signed rank test).

In SCG neurons, activation of PLC may lead to rises in intracellular Ca^2+^ when triggered by certain G_q_ coupled receptors (such as B_2_ bradykinin), but not by others (such as M_1_ muscarinic; Delmas et al., 2002). To reveal whether activation of P2Y_6_ receptors might elevate intracellular Ca^2+^, SCG neurons were transduced with the genetically encoded calcium indicator jGCamp8s (Zhang et al., 2021) using an adeno associated virus as vector. In 25 out of 43 transduced neurons tested, application of UDP led to a concentration dependent increase in cytosolic Ca^2+^ with half maximal effects in the range of 1 µM (Figures 4A–C).

In hippocampal neurons, rises in intracellular Ca^2+^ elicited by activation of P2Y_1_ receptors were found to trigger currents through K_Ca_2 channels (Schicker et al., 2010). To search for current responses in sympathetic neurons, UDP was applied to cells under voltage clamp at −70 mV, a value close to that measured in current clamp (see above). In quasi physiological solutions as used above, current responses to UDP were inwardly directed which is incompatible with K^+^ currents given a K^+^ equilibrium at < −100 mV. As it is well known that changes in neuronal excitability in response to elevations in cytosolic Ca^2+^ cannot only rely on an opening of Ca^2+^-activated K^+^-, but also of Ca^2+^-activated Cl^−^ channels (CaCC; Scott et al., 1995), subsequent current recordings were performed in the absence of K^+^ and with CsCl based bath as well as pipette solutions. In 12 out of 31 neurons tested, an inward current arose slowly during a 120 s exposure to UDP (1 µM). Upon removal of the nucleotide, current amplitudes gently declined towards baseline levels (Figure 4D). To test for a role of CaCCs, neurons were exposed to UDP twice with the second nucleotide application occurring in the continuing presence of the specific CaCC inhibitor CaCCInh-A01 (10 μM; Namkung et al., 2011). This drug significantly diminished current responses to UDP. Likewise, when neurons were exposed to 10 µM of the cell permeant Ca^2+^-chelator BAPTA-AM and the second UDP application was carried out in BAPTA-AM, the currents evoked by the nucleotide were lost. For comparison, when currents were triggered twice in the presence of solvent instead of CaCCInh-A01, their amplitudes did not decrease (Figures 4D–F). Thus, activation of P2Y_6_ receptors may lead to the opening of CaCCs in sympathetic neurons.

### 3.5 Roles of K_V_7 channel inhibition and CaCC activation in increased membrane excitability caused by Uridine diphosphate

**FIGURE 5:**
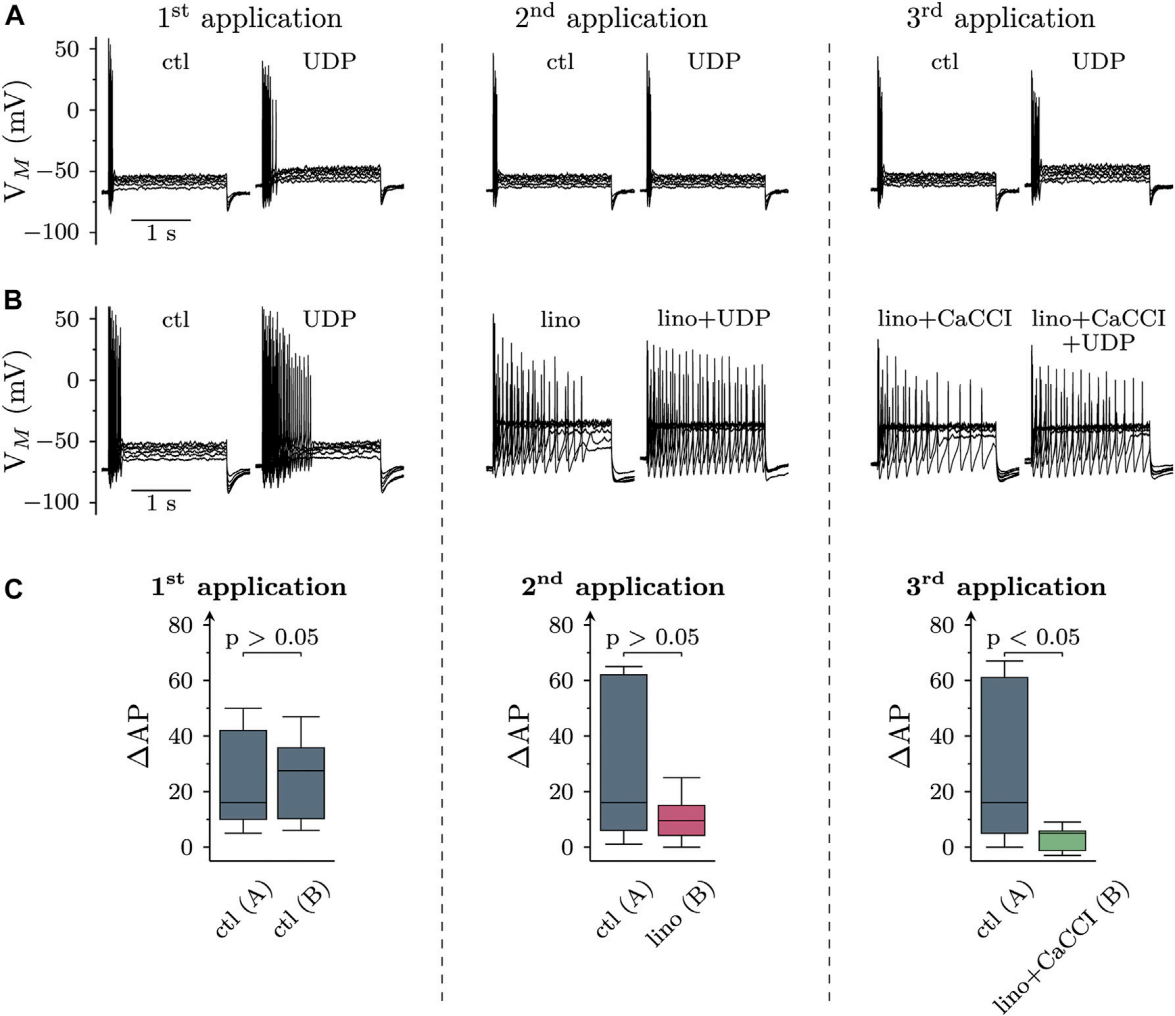
Roles of K_V_7 channel inhibition and CaCC activation in the effects of UDP on sympathetic neuron excitability. Membrane potential was measured in perforated-patch current clamp in single SCG neurons, and 6 depolarizing 2 s currents with amplitudes of 50, 100, 150, 200, 250, and 300 pA were injected to trigger action potentials. Three consecutive applications of UDP (10 µM for 120 s) were separated by two 5 min periods (represented by vertical dashed lines). **(A)** and **(B)** show original traces obtained before and during the presence of UDP, **(C)** summarizes the changes in AP firing (ΔAP) due to the presence of UDP with data at each left hand side derived from A and those at each right hand side derived from **(B)**. **(A)** Original current clamp traces were obtained with three consecutive applications of UDP (10 µM for 120 s) in solvent (DMSO) interleaved by 5 min periods of solvent (DMSO) only. The traces have been obtained just before and at the end of the three 120 s UDP applications. **(B)** Original traces were obtained with three consecutive applications of UDP (10 µM for 120 s), first in solvent (DMSO), and then in DMSO plus linopirdine (lino; 30 µM), and thereafter in DMSO plus linopirdine plus CaCCInh-A01 (10 μM; lino + CaCCI). These drugs or drug combinations were also present for 120 s prior to current injection. The traces have been obtained just before and at the end of the three 120 s UDP applications. Linopirdine on its own caused depolarizations that were compensated for by hyperpolarizing current injections. **(C)** Box plots showing changes in AP firing (ΔAP) in response to the three consecutive UDP applications (1st, 2nd, 3rd) either in solvent only [ctl; *n* = 11; as in **(A)** or in solvent followed by linopirdine (lino) and linopirdine plus CaCCI (lino + CaCCI; *n* = 12; as in **(B)**]. The *p* values above the three pairs of boxes indicate differences between solvent only (left hand boxes) and the drug treated groups (right hand boxes; Mann-Whitney *U*-test).

To reveal whether CaCC activation and/or K_V_7 channel inhibition might contribute to increased membrane excitability in response to P2Y_6_ receptor activation, cells were current clamped and exposed to 10 µM UDP for three 120 s periods interleaved by application of solvent. Under these conditions, UDP raised the numbers of action potentials fired in response to current injections (as described above, Figures 1A,B) to about the same extent during each of the three consecutive UDP application periods (Figures 5A,C).

To exclude K_V_7 channel inhibition from effects of UDP on membrane excitability, these channels were blocked by linopirdine (30 µM). This led to a depolarization by 2.1 (1.5–4.5) mV and raised the median number of action potentials triggered by current injections from 21.5 (16.3–32.5) to 30.0 (13.0–52.8) (*p* < 0.05; *n* = 12, Friedman test, Dunn’s multiple comparison). To compensate for the depolarization caused by Kv7 channel inhibition, a hyperpolarizing current was injected to bring the resting membrane potential back to pre-linopirdine levels as previously described in sensory neurons (Ray et al., 2019). Thereafter, with K_V_7 channels being blocked, the increase in action potential firing caused by the second UDP application declined as compared with the first UDP application. However, this increase in action potential firing due to UDP exposure in linopirdine was not different from that seen in solvent (Figure 5C). Subsequent addition of 10 µM CaCCInh-A01 failed to cause significant alterations in membrane potential (*p* > 0.05 Wilcoxon signed rank test) and action potential firing (*p* > 0.05; *n* = 12, Friedman test, Dunn’s multiple comparison), but further reduced the increase in excitability caused by the third UDP application as compared with the previous ones. With both, K_V_7 channels and CaCCs being blocked, the increase in action potential firing elicited by the final UDP application was significantly smaller than the analogous value obtained in solvent (Figure 5C). In analogy with linopirdine, when 10 µM CaCCInh-A01 was applied in the absence of this K_V_7 channel blocker, the median increase in action potential firing caused by UDP was 2.5 (2–3.75) and thereby not significantly different from the value of 4 (2–12.5) as observed in solvent beforehand (*p* > 0.05, Wilcoxon matched-pair signed rank test; *n* = 8).

**FIGURE 6:**
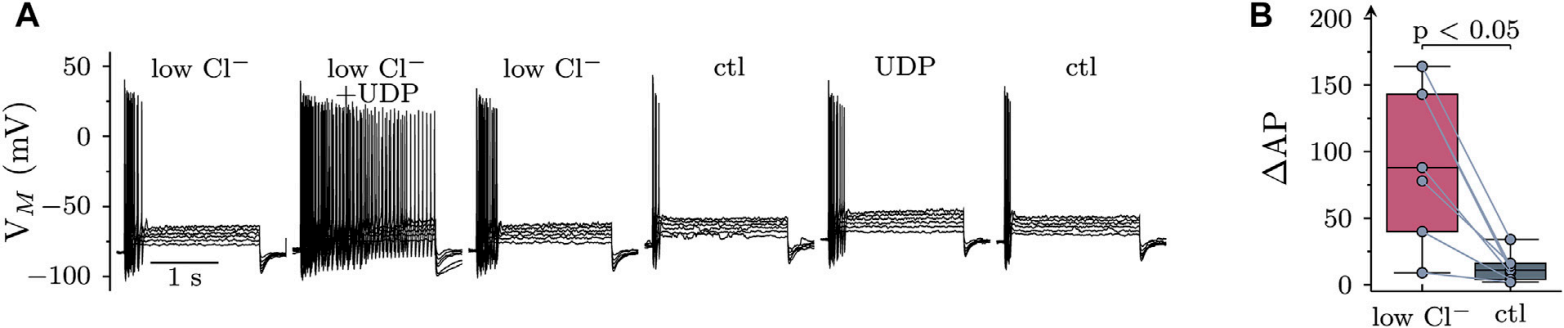
Role of extracellular chloride in the effects of UDP on sympathetic neuron excitability. Membrane potential was measured in perforated-patch current clamp in single SCG neurons, and 6 depolarizing 2 s currents with amplitudes of 50, 100, 150, 200, 250, and 300 pA were injected to trigger action potentials. **(A)** Original current clamp traces were obtained in a low Cl^−^ solution (89 mM; low Cl^−^), and current injection was performed before, during, and after application of UDP (1 µM for 120 s); thereafter, this protocol was repeated in standard Cl^−^ solution (149 mM). **(B)** Changes in AP firing in response to UDP in either low Cl^−^ or standard Cl^−^ (ctl) solution as shown in A (*n* = 7; Wilcoxon matched-pair signed rank test).

To obtain additional evidence for a contribution of chloride conductances to the effects of UDP, cells were current clamped in an external solution with reduced Cl^−^ concentrations (89 vs. 149 mM). Under these conditions, the reversal potential of Cl^−^ is at about −6 mV, while being at −19 mV under standard extracellular Cl^−^. This raises the Cl^−^ driving force and can be expected to augment UDP effects provided that Cl^−^ conductances are involved. In low extracellular Cl^−^, the membrane potential was hyperpolarized by 4.6 (2.8–7.5; *n* = 7) mV (Figure 6A). As shown in Figure 6, when UDP was applied in low Cl^−^, the resulting increase in excitability was significantly more pronounced than in standard conditions.

### 3.6 Uridine diphosphate provokes noradrenaline release in a CaCC-dependent manner

**FIGURE 7:**
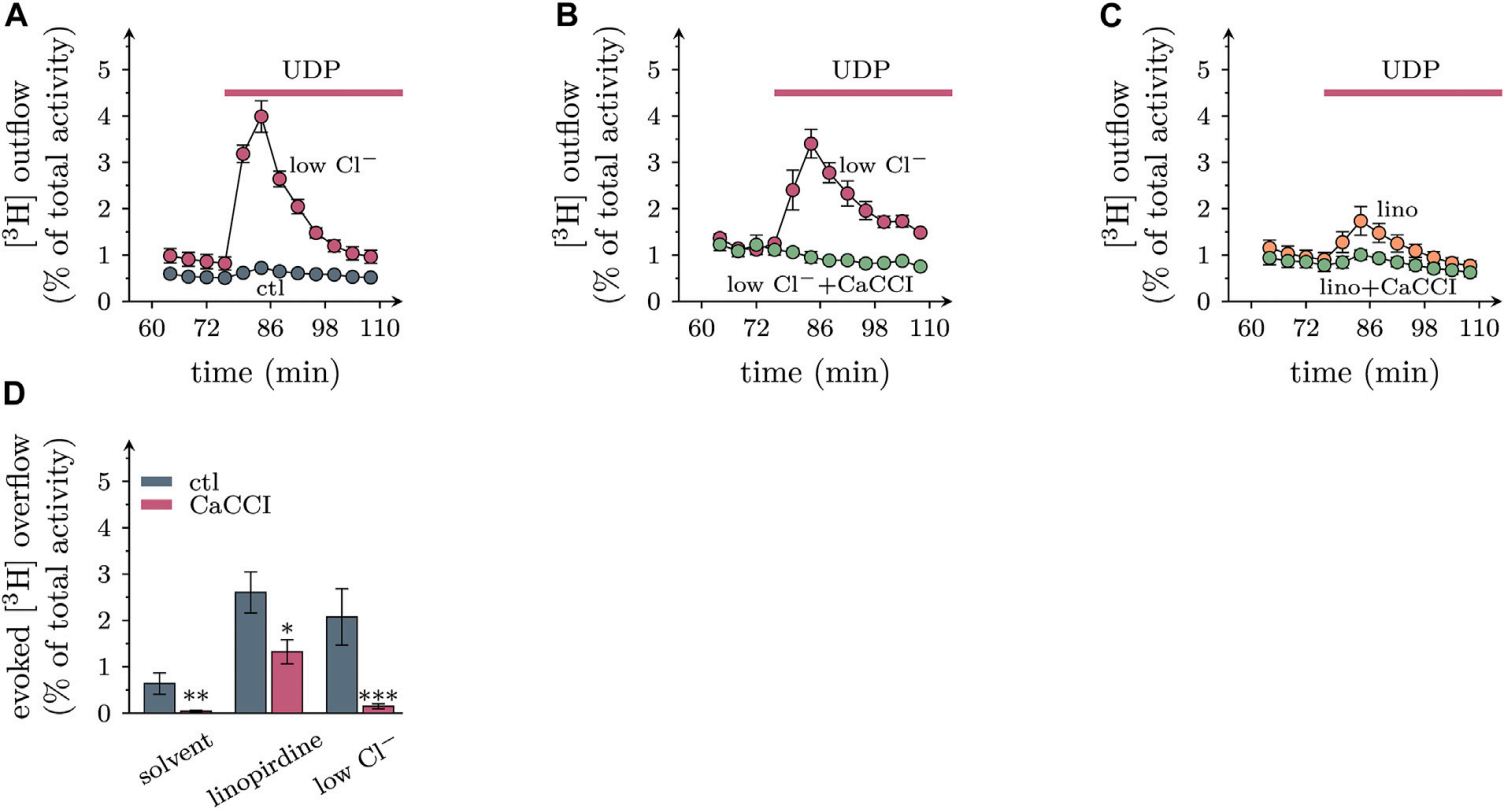
Roles of extracellular chloride and CaCCs in transmitter release from sympathetic neurons triggered by UDP. Primary cultures of rat SCG neurons were labeled with [^3^H]noradrenaline and superfused; subsequent to a 60 min washout period, 4 min fractions of superfusate were collected; UDP was present as indicated by the arrows. **(A)** to **(C)** Exemplary time course of [^3^H] outflow in solvent (DMSO), with 60 mM Cl^−^ being replaced by gluconate (low Cl^−^), and/or in presence of 30 µM linopirdine and/or 10 µM CaCCInh-A01 (CaCCI); *n* = 3 in each trace. **(D)** Effects of CaCCInh-A01 (10 μM; CaCCI) on [^3^H] overflow triggered by UDP in solvent, in 30 µM linopirdine, and with 60 mM Cl^−^ being replaced by gluconate (low Cl^−^); *n* = 9 for each bar. *, **, and *** indicate significant differences vs the respective control at *p* < 0.05, *p* < 0.01, and *p* < 0.001, respectively (pairwise Mann-Whitney tests).

To uncover whether stimulatory effects of UDP on membrane excitability might translate into altered transmitter output, cultures were labelled with [^3^H]noradrenaline, and outflow of previously incorporated radioactivity was quantified. Under continuous superfusion and after a 60 min washout period, small amounts of radioactivity were steadily delivered into the buffer. Exposure to 1 µM UDP slightly increased the rate of spontaneous tritium outflow, and this transient enhancement was largely augmented when 60 mM Cl^−^ in the buffer were replaced by gluconate (Figure 7A). UDP evoked tritium overflow was also seen when K_V_7 channels were blocked by 30 µM linopirdine (Figure 7C). Inclusion of CaCCI in the superfusion buffer diminished [^3^H] overflow triggered by UDP, be it under control conditions, in low Cl^−^, or in presence of linopirdine (Figure 7D). This indicates that CaCCs contribute to transmitter release from sympathetic neurons triggered by activation of P2Y_6_ receptors.

## 4 Discussion

### 4.1 Uridine diphosphate excites superior cervical ganglion neurons *via* P2Y_6_ receptors

In light of the key role of the sympathetic nervous system in the pathophysiology of cardiovascular diseases (Malpas, 2010), the recent emergence of P2Y_6_ receptors as drug targets in cardiovascular therapy (Burnstock, 2017; Zhou et al., 2020) has revived the interest in functions of that receptor subtype in sympathetic neurons. Even though uridine nucleotides had been reported to raise noradrenaline release from sympathetic neurons in primary culture, the receptors involved have remained unidentified and the underlying signaling cascades were uncovered only partially, if at all (Nörenberg et al., 2000; Vartian et al., 2001). Neuronal P2Y_1_, −2, −4, and −6 receptors have been shown to mediate an inhibition of K_V_7 channels (Hussl and Boehm, 2006), but UDP- or UTP-dependent noradrenaline release from sympathetic neurons did not appear to rely on this effector system (Nörenberg et al., 2000; Vartian et al., 2001). In the experiments described above, UDP was evidenced to increase membrane excitability and to inhibit currents through K_V_7 channels. Although this latter action can be expected to boost excitability (Brown and Passmore, 2009), the extent of K_V_7 channel inhibition did not show any correlation with the increase in membrane excitability (see Figure 3C). UDP-dependent action potential firing was reduced by the specific P2Y_6_ receptor antagonist MRS2578 (Mamedova et al., 2004), but not by the P2Y_2_ antagonist AR-C118925XX (Rafehi et al., 2017). This confirms the key role of the former receptor and renders a contribution of K_V_7 channels unlikely.

### 4.2 P2Y_6_ receptor activation excites superior cervical ganglion neurons by gating of CaCCs

As an inhibition of K_V_7 channels did not appear to be essential for the P2Y_6_ receptor mediated increase in membrane excitability, alternative ionic mechanisms were tested. Under conditions that favor chloride over other conductances, UDP turned out to induce slowly arising membrane currents that returned towards baseline values after removal of the nucleotide (see Figure 4D). Currents with similar kinetics in superior cervical ganglion neurons have been seen before upon activation of M_1_ muscarinic acetylcholine receptors (Salzer et al., 2014). The currents in response to both, UDP and the muscarinic agonist oxotremorine M (Salzer et al., 2014) were abolished by CaCCInh-A01, a potent and specific blocker of CaCCs (Hao et al., 2021).

The crucial role of Ca^2+^-dependent chloride conductances was corroborated further by independent experiments. First, fluorescence measurements of the genetically encoded calcium indicator jGCamp8s revealed that UDP raised intracellular Ca^2+^ with half maximal effects at 1 µM. The UDP-triggered increase in excitability displayed the same concentration dependence. Second, effects of UDP on membrane excitability were also tested after shifting the chloride equilibrium potential by more than 10 mV towards more positive values. This change in chloride equilibrium significantly reinforced the UDP dependent increase in excitability. Finally, the rise in excitability caused by UDP remained unchanged when K_V_7 channels were blocked by linopirdine. However, when linopirdine and CaCCInh-A01 were present at the same time, the UDP triggered augmentation in excitability was reduced significantly. Together, these results indicate that P2Y_6_ receptor activation excites sympathetic neurons through activation of CaCCs.

Considering that gating of CaCCs *via* P2Y_6_ receptors might be a relevant mechanism in the excitation of SCG neurons by UDP, leads to the question as to why an increase in chloride conductance might boost neuronal excitability. An explanation is provided by the fact that these neurons have intracellular chloride concentrations in the range of 30 mM (Ballanyi and Grafe, 1985) to 70 mM (Woodward et al., 1969) which results in chloride equilibrium potentials between approximately −40 and −20 mV in physiological extracellular chloride concentrations. Accordingly, activation of a chloride conductance must lead to a depolarization with an accompanying increase in excitability. In agreement with this conclusion, activation of GABA_A_ receptors in SCG neurons was reported to cause depolarization and to trigger noradrenaline release (Salzer et al., 2014).

Another emerging question is related to the nature of the relevant channel. In general, CaCCs are accepted as members of a family of “transmembrane proteins with unknown function,” in short TMEM 16, and also known as anoctamins (ANO). This family comprises Ca^2+^-dependent scramblases and ion channels, and most neuronal CaCCs have been identified as being TMEM16A/ANO1 (Falzone et al., 2018). Sympathetic neurons of the mouse have been shown to express ANO1 (Martinez-Pinna et al., 2018). On the basis of these considerations, we suggest that activation of P2Y_6_ receptors leads to an increase in excitability of sympathetic neurons *via* the gating of TMEM16A/ANO1.

### 4.3 P2Y_6_ receptor activation raises noradrenaline release by gating of CaCCs

Having established that chloride conductances play a key role in the effects of P2Y_6_ receptor activation on membrane excitability of sympathetic neurons, it remained to be demonstrated that the very same mechanisms were involved the actions of UDP on sympathetic transmitter release. As a first challenge of the role of chloride ions, the chloride equilibrium was shifted to more positive values as described above. As a consequence, [^3^H]noradrenaline efflux from SCG cultures in the presence of UDP was enhanced which can be used to argue in favor of a role of CaCCs. However, noradrenaline release triggered by UDP was also enhanced when K_V_7 channels were blocked by linopirdine. In this context, one has to take into account that linopirdine can facilitate noradrenaline release from SCG neuros when elicited by many secretagogue stimuli other than uridine nucleotides, such as activation of nicotinic acetylcholine or ATP P2X receptors and K^+^ depolarization. Hence, facilitation of stimulation-evoked noradrenaline release by linopirdine does by no means indicate that the respective stimulus relies on a modulation of K_V_7 channels (Kristufek et al., 1999).

The role of chloride conductances was corroborated further by the use of CaCCInh-A01. This TMEM16A/ANO1 blocker (Hao et al., 2021) significantly attenuated the secretory response to UDP whether evoked under control conditions, with the chloride equilibrium being shifted to more positive values, or with K_V_7 channels being blocked by linopirdine. These results permit the conclusion that activation of P2Y_6_ receptors facilitates noradrenaline release from sympathetic neurons *via* the gating of TMEM16A/ANO1.

### 4.4 Mechanisms and consequences of sympathoexcitation *via* P2Y_6_

Previous studies have concluded that UDP-evoked transmitter release from sympathetic neurons was largely independent of K_V_7 channels, but rather involved mechanisms relying on PKC (Nörenberg et al., 2000; Vartian et al., 2001). For the following reasons, this previous conclusion is not in contradiction with the present results: 1) cellular actions in response to P2Y_6_ receptor activation are known to involve PKC as exemplarily shown in microglia (Kataoka et al., 2011) and astrocytes (Kim et al., 2003). 2) Activation of PKC is sufficient to induce chloride currents in sympathetic neurons (Marsh et al., 1995). 3) Several PKC isoforms have been shown to mediate excitation of SCG neurons (Scholze et al., 2002). 4) Inhibitors of PKC prevent the gating of CaCCs *via* muscarinic M_1_ receptors (Salzer et al., 2014). Hence, it appears reasonable to assume that opening of TMEM16A/ANO1 due to P2Y_6_ receptor activation did involve PKC, even though this aspect was outside the focus of the present investigation.

Up to now, the pathophysiological relevance of P2Y_6_ receptors in cardiovascular disease has been associated with their functions in the immune system and the vasculature (Burnstock, 2017; Zhou et al., 2020). Overactivity of the sympathetic nervous system is another decisive step in the development of cardiovascular pathology, and many drugs provide cardiovascular protection by dampening sympathetic responses (Malpas, 2010). Accordingly, the present data provide evidence that P2Y_6_ receptor antagonists that are being developed for cardiovascular therapy (Zhou et al., 2020) may elicit some of their actions by interference with the sympathoexcitatory actions of these receptors. In addition, the results above point towards the role of TMEM16A/ANO1 in the signaling cascades of P2Y_6_ receptors in sympathetic neurons. This CaCC is also relevant for cardiovascular disease as its gating elevates blood pressure (Heinze et al., 2014). Hence, just as P2Y_6_ receptor antagonists may newly developed TMEM16A/ANO1 blockers (Hao et al., 2021) provide cardiovascular protection not only by actions on the vasculature, but also *via* the sympathetic nervous system.

### 4.5 Limitations of the study

In the vasculature, UTP is released from myocytes, endothelial cells, platelets, and white blood cells and is then degraded towards UDP, and UMP by various nucleotidases. These uridine nucleotides can then act in a paracrine manner on all the cellular components contained in the vascular system amongst which one can find a dense network of postganglionic sympathetic axons equipped with varicosities that serve to deliver sympathetic transmitter (Burnstock, 2017). Through this functional vessel architecture, UDP can be expected to act *in vivo via* the mechanisms described above in a pure *in vitro* system. As a major limitation, *in vivo* experiments have not been part of this study. Therefore, it remains open for future investigations whether CaCCs and/or K_V_7 channels of sympathetic neurons contribute to the cardiovascular effects of P2Y_6_ receptor ligands *in vivo* as detailed in the introduction.

## Data Availability

The raw data supporting the conclusion of this article will be made available by the authors, without undue reservation.
